# Recomendações e protocolos para o uso seguro de torniquetes em cirurgias de extremidades superiores e inferiores

**DOI:** 10.1055/s-0045-1809690

**Published:** 2025-07-29

**Authors:** Antonio Tufi Neder Filho, Túlio Vinícius de Oliveira Campos

**Affiliations:** 1 Departamento do Aparelho Locomotor, Faculdade de Medicina, Universidade Federal de Minas Gerais, Belo Horizonte, MG, Brasil; 2 Rede Mater Dei de Saúde, Belo Horizonte, MG, Brasil

**Keywords:** complicações, extremidade inferior, extremidade superior, protocolo, torniquetes, complications, lower extremity, protocols, tourniquets, upper extremity

## Abstract

O torniquete (TNQ) interrompe o fluxo sanguíneo para um determinado segmento anatômico e tem aplicação importante nas cirurgias ortopédicas ao proporcionar um campo operatório limpo de sangue. Os riscos e complicações atribuídos ao seu uso são aumento da dor, lesão por reperfusão, edema, trombose venosa profunda e lesão de nervos periféricos. As principais recomendações para se utilizar o TNQ e diminuir a ocorrência de complicações são: acolchoar adequadamente o membro; insuflar o torniquete até pressão 50 mmHg acima da pressão de perfusão para os membros superiores e 100 mmHg para os membros inferiores; evitar o uso em pacientes com caquexia, crianças, portadores de lúpus e coagulopatia; evitar manter o dispositivo insuflado por período superior a 2 horas; e ter equipe treinada e atenta para a desinsuflação, caracterizada pela possibilidade de sangramento, embolia pulmonar e síndrome metabólica mionefropática. Este artigo de atualização resume a melhor evidência acerca do emprego do TNQ nas cirurgias ortopédicas e propõe um protocolo para sua utilização segura.

## Abstract (en)

The tourniquet (TNQ) interrupts blood flow to a given anatomical segment and has critical applications in orthopedic surgeries by providing a blood-free operating field. The risks and complications attributed to its use are increased pain, reperfusion injury, edema, deep venous thrombosis, and peripheral nerve injury. The main recommendations for TNQ use and to reduce the occurrence of complications include adequate limb padding; TNQ inflation to pressures of 50 mmHg and 100 mmHg above the perfusion pressure for the upper and lower limbs respectively; avoid TNQ use in children and patients with cachexia, lupus, and coagulopathy; avoid keeping the device inflated for more than 2 hours; and have a trained team alert to deflation, which is characterized by the possibility of bleeding, pulmonary embolism, and myonephropathic metabolic syndrome. The present update article summarizes the best evidence on TNQ use in orthopedic surgeries and proposes a protocol for its safe use.

## Introdução

O torniquete (TNQ) é um aparelho que interrompe o fluxo sanguíneo para um determinado segmento anatômico. Ele pode ser utilizado para: proporcionar um campo operatório limpo de sangue; permitir a anestesia de um segmento pela técnica de Bier; facilitar a punção ao aumentar o ingurgitamento venoso no membro; e controlar o sangramento no trauma grave de extremidade. 1 2

Há estudos 3 que relatam os riscos relacionados ao TNQ, tais como: aumento da dor, lesão por reperfusão, edema, trombose venosa profunda (TVP), sofrimento nas bordas da ferida cirúrgica e lesão de nervos periféricos.

O conhecimento dos cirurgiões e da equipe de assistentes sobre a técnica de colocação, as contraindicações e o tempo máximo de permanência do TNQ em cirurgias ortopédicas é considerado inadequado. 4

O objetivo deste artigo é apresentar uma atualização sobre o uso do TNQ nas cirurgias ortopédicas e propor um protocolo baseado nesses achados para que o dispositivo seja utilizado de forma segura.

## Uso do torniquete nos membros superiores

As pressões de insuflação e o tempo de permanência do TNQ nas cirurgias dos membros superiores são inferiores àqueles empregados nas dos membros inferiores. A pressão máxima utilizada é de 250 mmHg, e o tempo médio dos procedimentos é de 30 minutos. O manguito pode ser posicionado no braço ou no antebraço; este último posicionamento pode provocar a flexão dos dedos para a região palmar e atrapalhar a visão do campo a ser operado. 2

**Fig. 1:**
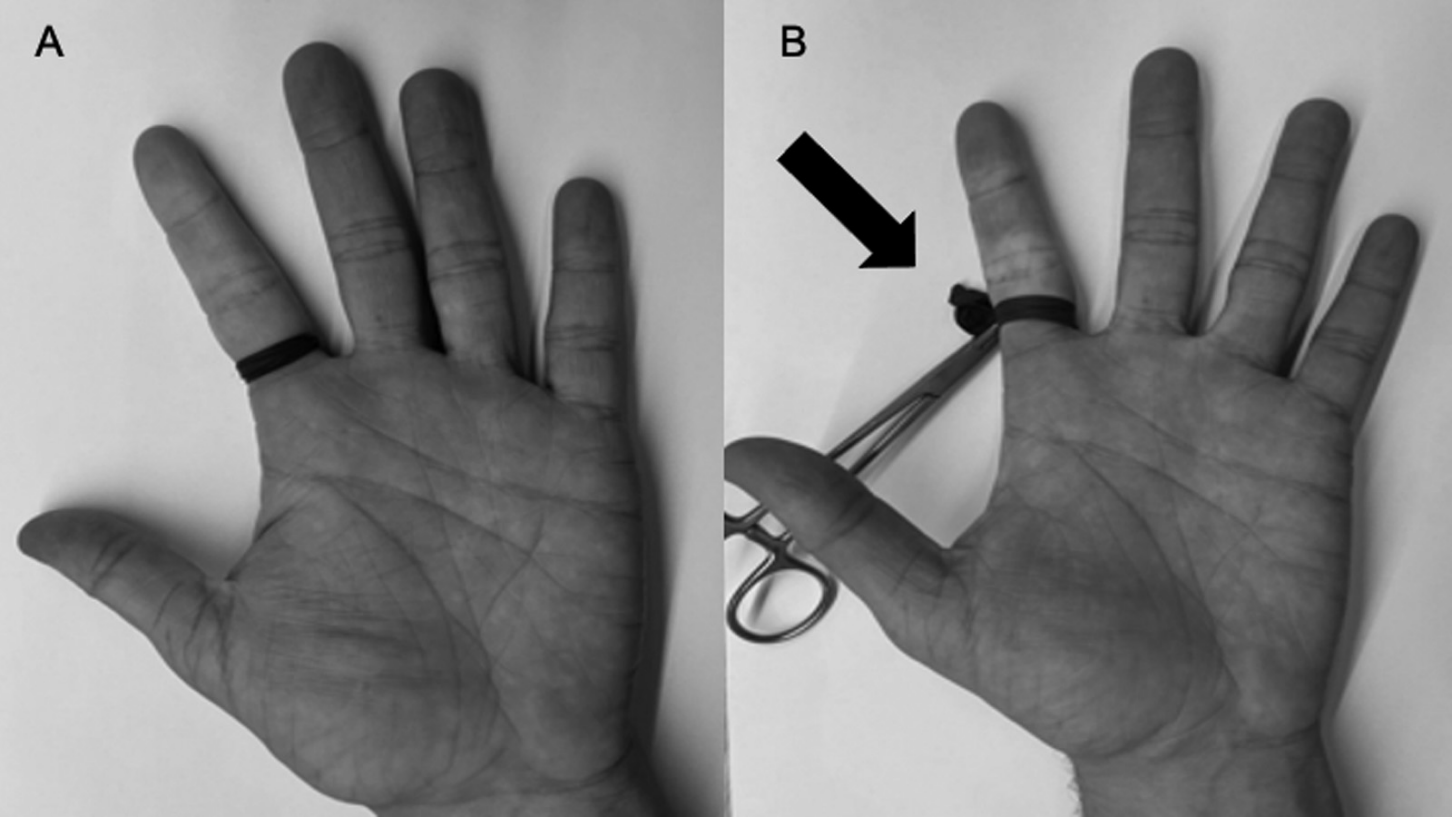
( **A** ) Torniquete elástico aplicado na base do segundo dedo da mão. A pressão desses dispositivos não é controlada, e eles devem ser coloridos para se evitar o esquecimento. ( **B** ) Dedo de luva aplicado na base do dedo. A utilização de uma pinça dificulta o esquecimento e a lesão iatrogênica da extremidade (seta preta).

A pressão capilar nos membros superiores é menor quando comparada à dos membros inferiores; por isso, a pressão necessária para a insuflação do TNQ é menor nos primeiros. 5 Um tipo específico de TNQ dos membros superiores é o aplicado nos dedos da mão. Neste caso, bandas elásticas coloridas ou um dedo de luva na base do dedo são utilizados. A desvantagem desse tipo de TNQ é que a pressão utilizada não pode ser controlada. Recomenda-se prendê-lo na base do dedo com uma pinça. Ao fazer isso, evita-se o seu esquecimento e a amputação iatrogênica do segmento ( Fig. 1 ). 2

Estudos comparativos 2 não mostraram diferença entre vários métodos para a exsanguinação dos membros superiores, tais como elevação do membro, esvaziamento utilizando faixa elástica de Esmarch, ou drenagem manual. Os desfechos avaliados foram capacidade de fornecer um campo limpo e repercussões hemodinâmicas. A comparação entre a utilização dos TNQs de silicone e os pneumáticos também não apresentou resultados conclusivos. Defende-se que o gradiente de pressão nos limites do TNQ é menor nos de silicone, que automaticamente fazem uma exsanguinação do membro e têm as bordas mais arredondadas. 2

As lesões de nervo relacionadas ao TNQ são 2,5 vezes mais frequentes nos membros superiores, sendo o nervo radial o mais acometido. Atribui-se esse aumento de risco ao envoltório reduzido de partes moles nesta região. 2

## Uso do torniquete nos membros inferiores

Há controvérsias sobre o emprego do TNQ nas cirurgias de membros inferiores. O maior argumento a favor destes dispositivos é a praticidade de se operar com um campo limpo e a redução da perda intraoperatória de sangue. Os pontos negativos incluem aumento de 1 ponto no valor atribuído à dor na escala visual analógica (EVA), aumento de 3 mg no consumo de opioide no primeiro dia após a cirurgia e perda transitória de força no segmento em que foi utilizado. 2

Em uma revisão sistemática e metanálise, Zhang et al. 6 avaliaram o emprego do TNQ em cirurgias artroscópicas dos membros inferiores; os autores não identificaram diferença na qualidade da visibilização do campo operatório, nem no tempo cirúrgico. Além disso, salientaram 6 que o uso do TNQ está relacionado à fraqueza muscular no pós-operatório e modifica parâmetros intraoperatórios, como a mobilidade da patela nos procedimentos de estabilização femuropatelar. Wang et al., 7 em revisão sistemática e metanálise que incluiu pacientes submetidos a artroscopia simples e reconstruções ligamentares, avaliaram 16 estudos com 1.132 participantes, e concluíram que o grupo sem TNQ apresentou menor perda sanguínea no pós-operatório e menor consumo de analgésicos. Não foi identificada diferença na visibilização cirúrgica, na dor, na força do quadríceps, nem no tempo cirúrgico.

Na cirurgia de reconstrução do ligamento cruzado anterior, o emprego do TNQ foi associado a maior atrofia do quadríceps, fraqueza e alterações na eletroneuromiografia nas primeiras 4 e 12 semanas de pós-operatório. Não houve diferença na avaliação dos pacientes 1 ano após a cirurgia. 8 9

O emprego do TNQ na artroplastia total do joelho é um dos temas mais discutidos na literatura recente. Os trabalhos discutem os benefícios e os riscos relacionados à sua utilização, o momento de insuflação/liberação e os efeitos nos curto e longo prazos. A análise dos estudos deve considerar o momento em que estes foram conduzidos. A inclusão do ácido tranexâmico, a introdução das próteses não cimentadas e da robótica nas cirurgias de substituição articular interferiu nos desfechos críticos, como perda sanguínea; por isso, é importante avaliar o contexto em que a artroplastia foi realizada antes de fazer uma comparação entre os estudos.

Em uma revisão sistemática e metanálise, Zhang et al. 10 compararam a liberação do TNQ antes e depois do fechamento da incisão nas artroplastias do joelho. Os autores 10 incluíram 1.010 pacientes e demonstraram que não houve diferenças significativas na perda sanguínea calculada, na perda sanguínea pós-operatória, na queda de hemoglobina, na taxa de transfusão, nas complicações maiores e na TVP. O grupo em que o TNQ foi liberado antes do fechamento da ferida apresentou maior volume de perda sanguínea total e tempo cirúrgico. Entretanto, liberar o TNQ antes do fechamento da ferida pode reduzir as complicações menores. 10

Han et al., 3 em uma revisão sistemática e metanálise que incluiu 29 ensaios clínicos randomizados e 2.512 artroplastias do joelho, concluíram que o TNQ diminuiu a perda sanguínea intraoperatória e o tempo cirúrgico e aumentou a interdigitação do manto de cimento. Não houve diferença no volume total de sangue perdido, na taxa de hemotransfusão, nem na taxa de TVP. Por outro lado, a utilização do TNQ está associada ao aumento da dor no pós-operatório, a uma piora da amplitude de movimento e dos escores funcionais, e ao aumento do edema e do tempo de permanência hospitalar. 3 Lu et al., 11 em revisão sistemática e metanálise, também encontraram maior penetração do manto de cimento e salientaram a importância deste achado na longevidade dos implantes. Os autores 11 não encontraram diferença na perda sanguínea, mas houve maior ocorrência de dor nos pacientes em que o TNQ foi utilizado.

Sun et al., 12 em uma metanálise, avaliaram 14 ensaios clínicos randomizados com 1.720 artroplastias totais do joelho realizadas com ácido tranexâmico. Os autores 12 compararam pacientes que utilizaram ou não TNQ no procedimento, e concluíram que o dispositivo apresentou correlação com menor perda sanguínea intraoperatória e duração da cirurgia, mas também, correlação com perda sanguínea oculta maior e amplitude de movimento diminuída. Nenhum dos outros parâmetros avaliados foram diferentes entre os grupos: hemoglobina, perda total de sangue, taxa de transfusão, volume de drenagem, pontuação na EVA no dia da cirurgia ou 1, 2, 3, 5, 7 ou 30 dias após a cirurgia, pontuação no escore do Hospital for Special Surgery (HSS) em 7 dias, ou 1,3, ou 6 meses após a cirurgia, circunferência do joelho, duração da internação hospitalar e complicações como TVP e infecção. 12 He et al. 13 compararam pacientes submetidos à artroplastia do joelho que utilizaram o TNQ durante todo o ato cirúrgico com aqueles em que o dispositivo foi acionado apenas no momento da cimentação. Os autores 13 concluíram que o uso restrito do TNQ apenas na cimentação diminuiu o volume do dreno no pós-operatório e aumentou a perda sanguínea total sem interferir na taxa de hemotransfusão; desse modo, recomendaram o uso do TNQ em tempo integral.

Ahmed et al., 14 em revisão da Cochrane, concluíram que o TNQ aumentou a incidência de eventos adversos na artroplastia total do joelho (risco relativo: 1,73) e aumentou a dor no primeiro dia de pós-operatório. Em relação ao último achado, a diferença foi de apenas 1 ponto na EVA, de modo que foi considerada irrelevante no cenário prático. Os autores 14 recomendaram que, no caso da utilização do dispositivo pela equipe cirúrgica, o paciente deve ser informado sobre o risco aumentado de complicações.

Horlocker et al. 15 avaliaram pacientes submetidos à revisão de artroplastia total do joelho com TNQ (tempo cirúrgico médio: 145 minutos). A taxa de paralisia dos nervos tibial e/ou peroneal foi de 7,7%. Houve recuperação completa em 100% das paralisias do nervo tibial e em 89% das paralisias do fibular. Os autores 15 avaliaram o subgrupo de procedimentos com tempo de TNQ maior do que 180 minutos: houve menor incidência de complicações neurológicas nos pacientes com intervalo de deflação do dispositivo maior do que 30 minutos (22%) em comparação aos pacientes sem intervalo (42%) ou com intervalo menor do que 30 minutos (39%).

Os estudos que avaliam o uso do TNQ no tratamento de fraturas são limitados e apresentam inúmeros questionamentos relacionados à sua metodologia. Præstegaard et al., 16 em revisão sistemática sobre o uso do TNQ no tratamento de fraturas, não identificaram benefícios relacionados à utilização deste dispositivo na cirurgia do trauma.

## Riscos e complicações

As complicações atribuídas ao uso do TNQ podem se manifestar no intraoperatório e no pós-operatório.

No intraoperatório, a maior preocupação ocorre no momento da liberação da pressão do TNQ. Neste momento, a equipe deve ser avisada e ficar atenta para as complicações mais comuns. A possibilidade de desprendimento de um grande trombo venoso do membro exige um adequado monitoramento pela equipe de anestesiologia. O uso do TNQ não é um fator de risco independente para trombose; todavia, o paciente que normalmente recebe esse dispositivo no seu procedimento apresenta um risco maior pela natureza desse procedimento. Outra complicação que pode ocorrer neste momento é a “síndrome metabólica mionefropática”, caracterizada por acidose metabólica, hipercalemia, mioglobulinemia, mioglobinúria e disfunção renal. Paradoxalmente, a liberação do TNQ está relacionada a um aumento da atividade trombolítica, à ativação das vias da antitrombina III e da proteína C, que podem ser implicados no aumento do sangramento que ocorre logo após a liberação do TNQ. 17

A maioria dos estudos sobre os efeitos do TNQ nos músculos utilizaram modelos animais com pressão de cerca de 350 mmHg por 2 horas. Os estudos 5 revelaram dois tipos de lesão muscular possíveis: compressão direta pelo TNQ e lesão isquêmica distal. O maior efeito na força muscular ocorreu nos primeiros dias após o uso, com recuperação de mais de 80% da força após 2 semanas. Os autores 5 relataram uma maior resistência dos nervos à lesão aguda; no entanto, quando esta ocorre, a recuperação é mais lenta quando comparada à da lesão muscular.

Além disso, parestesias e paralisias temporárias ou definitivas são atribuídas ao uso do dispositivo em 0,024% dos casos, e podem ter relação com a utilização de aparelhos descalibrados. Estudos 1 mostram que de 35% a 65% dos aparelhos utilizados não estão devidamente calibrados. Clinicamente, a incidência de lesão dos nervos fibular e tibial é maior nos pacientes que permanecem com o TNQ insuflado além de 150 minutos. 15

A embolia pulmonar pode ocorrer durante o esvaziamento do membro. Tal complicação é mais comum nas cirurgias do trauma; todavia há relatos 1 em procedimentos eletivos. Portanto, o momento da liberação do TNQ deve ser comunicado a todos os profissionais que assistem o paciente no peroperatório. 1

Nas crianças, o TNQ eleva a frequência cardíaca, a pressão arterial e a temperatura corporal central, por reduzir a área de troca de calor. As complicações classicamente descritas para os adultos, como dor, edema do membro, síndrome compartimental, lesões de pele por pressão ou queimadura química também foram descritas para crianças. Reilly et al. 18 utilizaram a medida da pressão de oclusão do membro como parâmetro para definir a insuflação do TNQ em cirurgias artroscópicas do joelho de crianças. As pressões utilizadas foram em média de 151 mmHg. Neste estudo, os autores 18 observaram um grupo de pacientes em que a pressão de insuflação foi arbitrariamente definida como de 300 mmHg. Não foram relatadas complicações, e o efeito do TNQ na visibilização do campo operatório foi adequado em ambos os grupos. A recomendação da pressão de insuflação do TNQ na população infantil é variável; todavia, autores 18 19 relatam bom efeito na visibilização do sítio cirúrgico a partir de 25 mmHg acima da pressão de perfusão para membros superiores, e de 50 mmHg para membros inferiores.

**Fig. 2:**
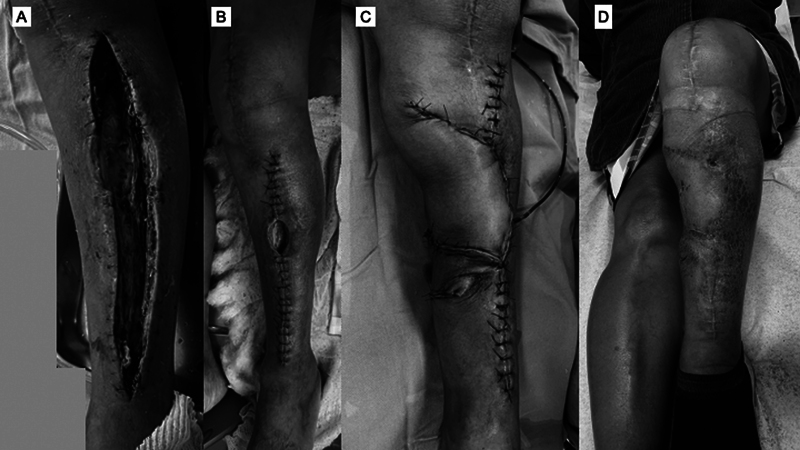
Paciente com quadro de síndrome compartimental aguda em pós-operatório imediato de artroplastia total do joelho. O torniquete é uma das justificativas encontradas para essa complicação. ( **A** ) Fasciotomia; ( **B** ) fechamento da fasciotomia e defeito de fechamento na região anterolateral da perna; ( **C** ) retalho miofasciocutâneo do gastrocnêmio medial para fechamento da lesão; e ( **D** ) aspecto final aos 3 meses de seguimento.

Lesões arteriais ocorrem principalmente nos pacientes com doença arterial prévia, e se apresentam clinicamente na forma de trombose. A síndrome compartimental aguda é descrita e atribuída ao uso do TNQ por tempo prolongado, utilização de pressão muito elevada e descalibração do aparelho ( Fig. 2 ).

Haladdin et al., 20 em uma revisão sistemática, avaliaram qualitativamente o efeito das intervenções anestésicas, o uso de antioxidantes e o efeito do pré-condicionamento no estresse oxidativo relacionado ao uso dos TNQs. Todos os cinco estudos que avaliaram o emprego do propofol e os três que avaliaram o pré-condicionamento demonstraram um efeito benéfico dessas ações no estresse oxidativo. Os autores 20 salientam a baixa qualidade da evidência existente sobre o tema e a ausência de estudos sobre as repercussões clínicas das intervenções, e propõem um estudo do efeito clínico desses agentes como linha de pesquisa.

A Tabela 1 resume as principais diferenças na aplicação dos TNQs nos membros superiores e inferiores.

**Tabela 1:** Configuração para a aplicação e complicações relacionadas ao uso dos torniquetes nos membros superiores e inferiores

	Membro superior	Membro inferior
**Pressão de insuflação máxima**	250 mmHg	300 mmHg
**Pressão de insuflação**	50 mmHg acima da pressão de perfusão	50 mmHg acima da pressão de perfusão
**Complicações**	• Esquecimento (dedos); e • Lesão de nervo periférico (menor envoltório de partes moles)	• Trombose; e • Síndrome de reperfusão

## Protocolo sugerido para a aplicação dos torniquetes

O protocolo a seguir organiza e resume as informações coletadas dos artigos 1 2 5 21 22 23 utilizados para escrever este artigo de atualização. Os momentos-chave para a aplicação do TNQ foram divididos em: 1) preparo do membro; 2) características do TNQ; 3) tempo de isquemia; e 4) pressão.

## Preparo do membro

A pele abaixo do TNQ deve ser protegida por bandagens de lã, algodão ou tecido sintético; todavia, o número de camadas não deve ser maior do que dois. À medida que se aumenta o número de camadas de tecido, a eficácia do TNQ é reduzida. 1

Deve-se ter cuidado para evitar o acúmulo de solução antisséptica, principalmente álcool, embaixo do TNQ. Essa ocorrência pode causar lesões de pele, principalmente em idosos e crianças. Para evitá-la, o isolamento com plástico ou compressas pode ser útil. 1

Antes de insuflar o TNQ, é importante elevar o membro por cerca de 2 minutos para esvaziar o membro de sangue. Outra opção é utilizar bandagens compressivas. A insuflação do TNQ deve ser rápida, pois, primeiramente, ocorre interrupção do fluxo venoso e, depois, do arterial. Se o tempo entre esses marcos for elevado, ocorrerá aumento da pressão venosa, e o efeito do TNQ pode ser paradoxal no sangramento. 1 2

Outro detalhe importante é administrar o antibiótico profilático antes de insuflar o manguito, idealmente antes de iniciar o preparo do membro, 1 hora antes da incisão de pele. 5

## Características do torniquete

A largura do manguito apresenta correlação com a eficácia do dispositivo. A relação da largura do manguito com a circunferência do membro é importante para definir a pressão de oclusão. Sabe-se que manguitos muito estreitos requerem pressões de insuflação maiores para serem eficazes. Autores 21 identificaram que, quando a relação entre a largura do manguito e o diâmetro do membro é maior do que 0,3:1, a pressão para oclusão da perfusão é menor do que a sistólica, o que pode reduzir o risco de lesões provocadas pelo TNQ.

**Fig. 3:**
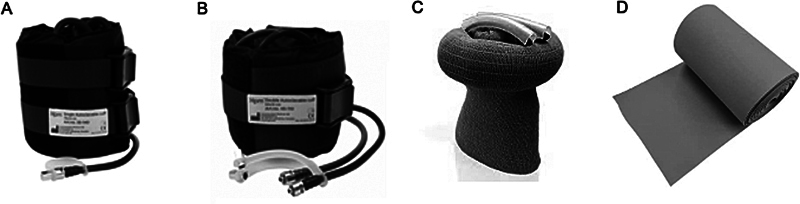
Tipos de torniquete disponíveis. ( **A** ) Torniquete simples pneumático. ( **B** ) Torniquete duplo, que permite alternar os pontos de insuflação. ( **C** ) Torniquete de silicone estéril. ( **D** ) Faixa de Esmarch.

A maioria dos TNQs são pneumáticos, não estéreis e podem ser utilizados inúmeras vezes. Porém, há dispositivos de silicone que são estéreis e de uso único. A vantagem dos últimos é a possibilidade de atingir locais de difícil acesso, como a raiz da coxa, e permitir a fixação de fraturas diafisárias do fêmur com haste retrógrada, por exemplo ( Fig. 3 ).

## Tempo de isquemia

Em indivíduos com idade inferior a 50 anos, o limite de tempo de 2 horas de isquemia é justificado pela acidose que ocorre no segmento venoso e suas possíveis repercussões. O intervalo de 30 minutos após 2 horas da aplicação do TNQ é proposto por alguns autores para estender o seu tempo de uso. O resfriamento do membro é outro fator que reduz os efeitos da isquemia e pode ser empregado nos procedimentos com previsão de duração superior a 2 horas. Para cirurgias com duração mais prolongada, o emprego de manguitos duplos permite alternar a área de compressão. 1

## Pressão

Em relação à pressão máxima a ser aplicada, os autores de um artigo de revisão 5 da American Academy of Orthopaedic Surgeons (AAOS) preconizam a utilização da pressão máxima de 250 mmHg para cirurgias dos membros superiores, e de 300 mmHg para membros inferiores.

Outro parâmetro que pode ser utilizado é a pressão de oclusão do membro; calculada com auxílio do Doppler, é definida como a pressão necessária para interromper o fluxo sanguíneo para aquele segmento anatômico. Esse processo é recomendado principalmente para pacientes com doença arterial grave e calcificação da parede das artérias, nos quais se pode superestimar a pressão necessária para interromper o fluxo arterial para o membro. 2

Ding et al., 22 em UMA metanálise publicada em 2019, concluíram que a definição da pressão de insuflação do TNQ baseada na pressão arterial sistólica medida POR Doppler ou oxímetro de pulso associados à circunferência do membro reduz o valor de insuflação do manguito, melhora o efeito hemostático e reduz a incidência de complicações.

Há uma preocupação muito grande dos cirurgiões e anestesistas em relação ao tempo de permanência do TNQ; todavia, a pressão aplicada é também fator importante nesse processo. A pressão aplicada no TNQ está relacionada à lesão física nos nódulos de Ranvier, fator importante para a ocorrência de complicações. 23

**Fig. 4:**
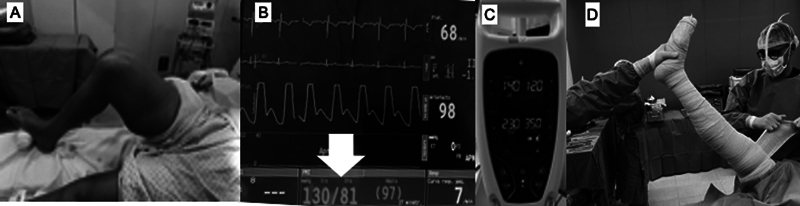
Etapas principais a serem observadas na colocação do torniquete para cirurgias ortopédicas. ( **A** ) manguito posicionado na raiz do membro com proteção da pele por ataduras de crepom. ( **B** ) mensuração da pressão arterial sistólica do paciente (seta branca). ( **C** ) insuflação do manguito 100 mmHg além da pressão sistólica ou da pressão de perfusão do membro. ( **D** ) esvaziamento do membro por atadura compressiva antes da insuflação do torniquete. Após essa sequência, a equipe cirúrgica deve anotar no quadro do *time out* a hora da insuflação do manguito, que deve permanecer pelo período máximo de 120 minutos.

A Fig. 4 ilustra os passos mais importantes que devem ser seguidos na aplicação dos TNQs nas cirurgias ortopédicas: 1) posicionamento do TNQ; 2) definição da pressão de insuflação; e 3) preparo do membro.

**Table:** 

Pontos-chave no emprego do TNQ nas cirurgias ortopédicas
***Antes de insuflar o TNQ***
Elevar o membro para realizar o seu esvaziamento.
Administrar antibiótico profilático antes de insuflar e 1 hora antes da incisão.
Acolchoamento adequado sob o manguito.
Definir a pressão de insuflação: 50 mmHg acima da pressão de perfusão para membros superiores, e 100 mmHg para membros inferiores
Tomar cuidado com pacientes especiais: aqueles com caquexia, portadores de lúpus eritematoso sistêmico, coagulopatia e crianças. Avaliar a utilização caso a caso.
**Após insuflar o TNQ**
Evitar tempo de isquemia superior a 2 horas.
Ter equipe de apoio treinada para que os tempos de insuflação/esvaziamento sejam respeitados: máximo de 2 horas, seguidas de pelo menos 30 minutos de reperfusão.
**Antes de desinsuflar o TNQ**
Comunicar a equipe de apoio na sala cirúrgica, principalmente a de anestesiologia, para que atenção seja dada para as repercussões agudas: 1) embolia pulmonar; 2) síndrome metabólica mionefropática; e 3) sangramento.

## Conclusão

O uso do TNQ torna o procedimento cirúrgico mais rápido e seguro; porém, todos os pontos discutidos neste protocolo devem ser observados para a obtenção dos seus benefícios e minimizar ao máximo os seus riscos.
